# Supplementary material for: Determining the structure of protein-bound ceramides, essential lipids for skin barrier function
Source: iScience. 2023 Oct 18;26(11):108248. doi: 10.1016/j.isci.2023.108248 (PMC10641502; doi:10.1016/j.isci.2023.108248)
Supplement: Document S1. Figures S1‒S7 and Table S1 [file mmc1.pdf]

iScience, Volume *26*

Supplemental information

Determining the structure of protein-bound ceramides, essential lipids for skin barrier function

Yusuke Ohno, Tetsuya Nakamura, Takafumi Iwasaki, Akira Katsuyama, Satoshi Ichikawa, and Akio Kihara

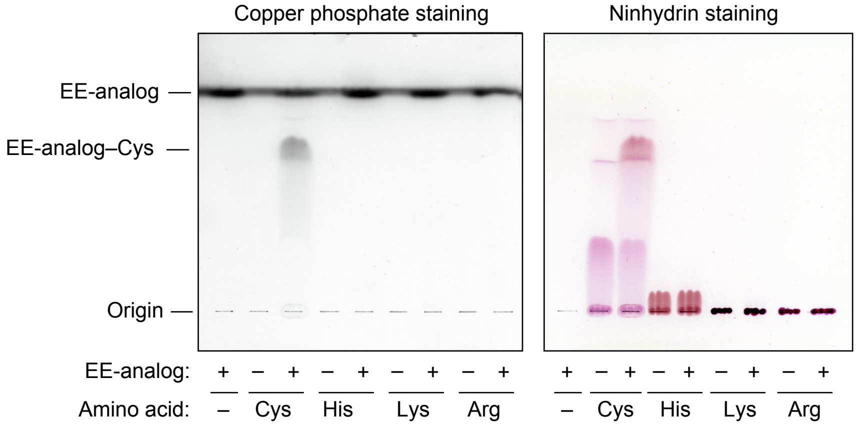

Figure S1. Conjugate formation in the absence of water, related to Figure 2. EE-analog (2 mM, dissolved in CH_3OH) was mixed with an amino acid (Cys, Ser, His, Arg, or Lys; 2 mM each, dissolved in CH_3OH) and incubated at 37 °C for 1 h. The reaction products were separated via TLC using $CHCl_3/CH_3OH$ (1:2, v/v) as a developing solvent and visualized using copper phosphate staining (left) or ninhydrin staining (right).

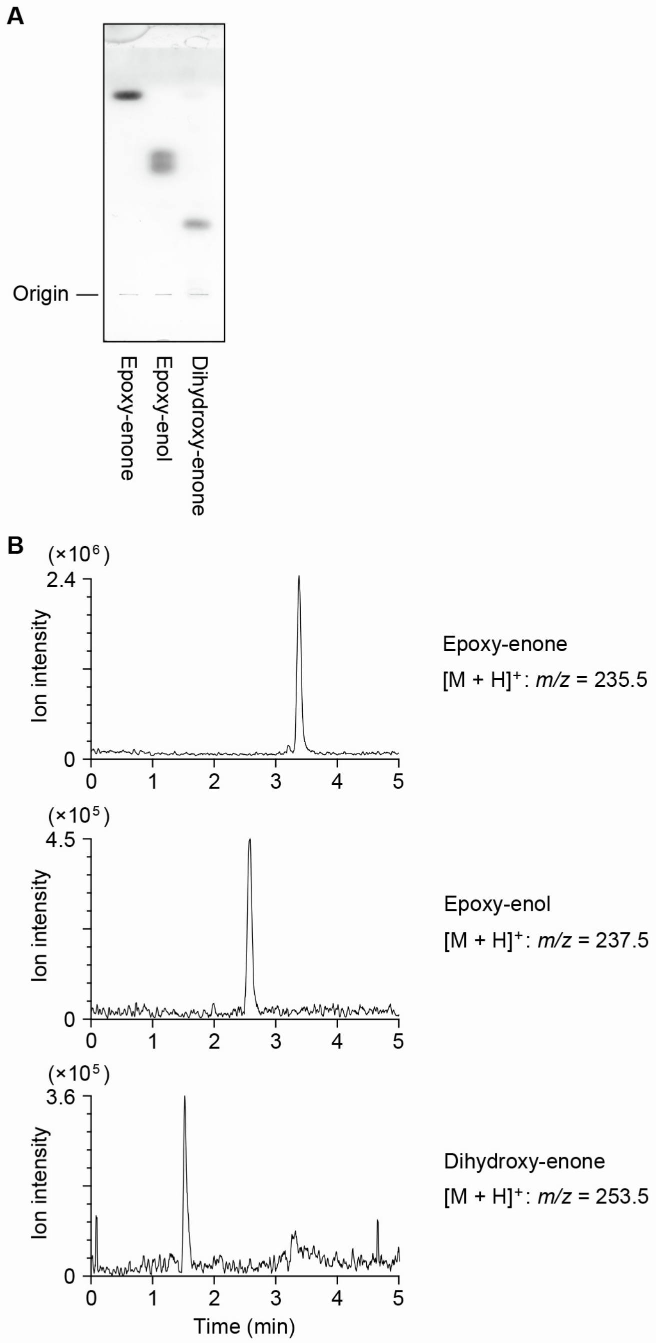

Figure S2. Detection of EE-analog and its derivatives, related to Figure 3.
(A) EE-analog and its derivatives were separated by TLC and subjected to copper phosphate staining.
(B) The molecular ions with *m/z* = 235.5, 237.5, and 253.5 were detected via LC-MS using single ion monitoring mode.

A

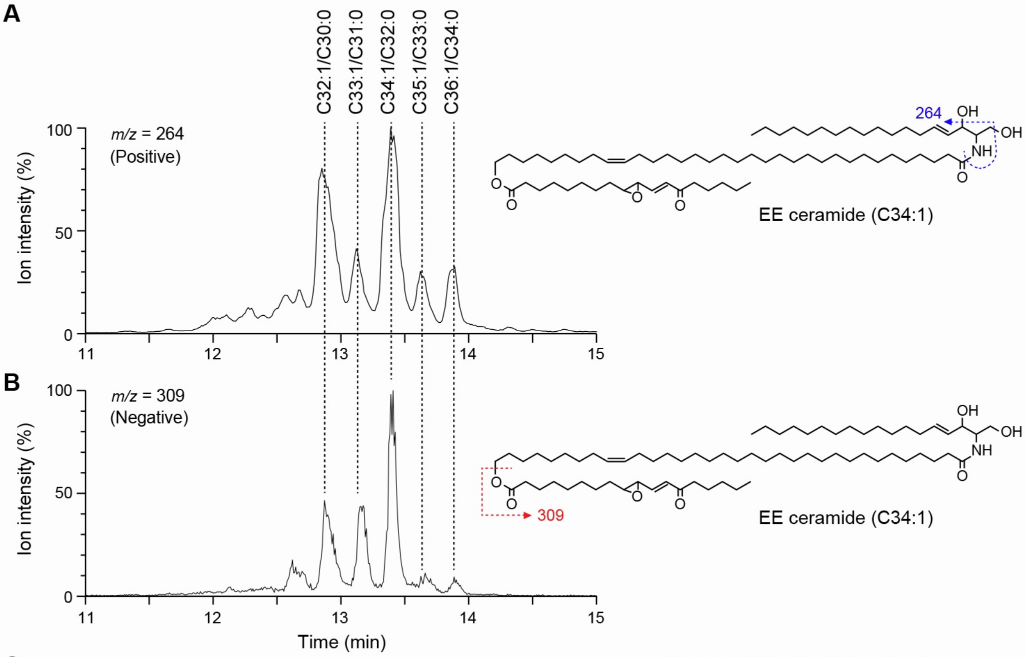

C

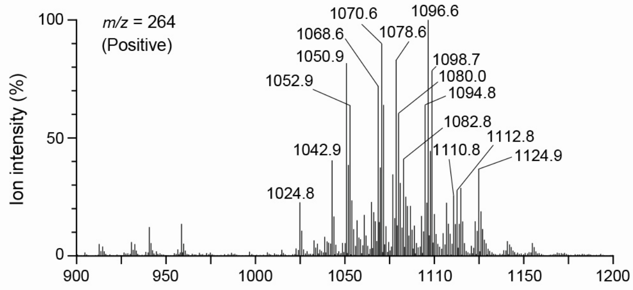

[M + H – H_2O]$^+$	[M + H]$^+$	*N*-acyl
1022.8	1040.8	C30:1
1024.8	1042.8	C30:0
1036.8	1054.8	C31:1
1038.8	1056.8	C31:0
1050.8	1068.8	C32:1
1052.8	1070.8	C32:0
1064.8	1082.8	C33:1
1066.8	1084.8	C33:0
1078.8	1096.8	C34:1
1080.8	1098.8	C34:0
1092.8	1110.8	C35:1
1094.8	1112.8	C35:0
1106.8	1124.8	C36:1
1108.8	1126.8	C36:0

D

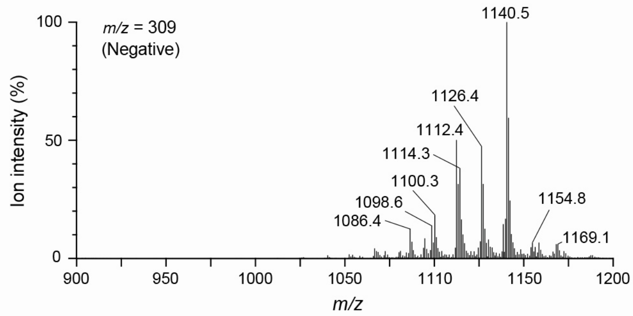

[M + HCOO]$^-$	*N*-acyl
1084.8	C30:1
1086.8	C30:0
1098.8	C31:1
1100.8	C31:0
1112.8	C32:1
1114.8	C32:0
1126.8	C33:1
1128.8	C33:0
1140.8	C34:1
1142.8	C34:0
1154.8	C35:1
1156.8	C35:0
1168.8	C36:1
1170.8	C36:0

Figure S3. Detection of EE ceramides by LC-MS/MS, related to Figure 4.
EE ceramides were extracted from the protein-bound ceramide fractions and subjected to LC-MS/MS analysis using precursor ion scanning mode (scan range of *m/z*, 900–1,200) to detect precursor ions with the product ion of *m/z* = 264 (positive ion mode) (A and C) and those of *m/z* = 309 (negative ion mode) (B and D). Total ion current chromatograms (A and B) and the mass spectra of the retention time of 12–14 min (C and D) are shown.

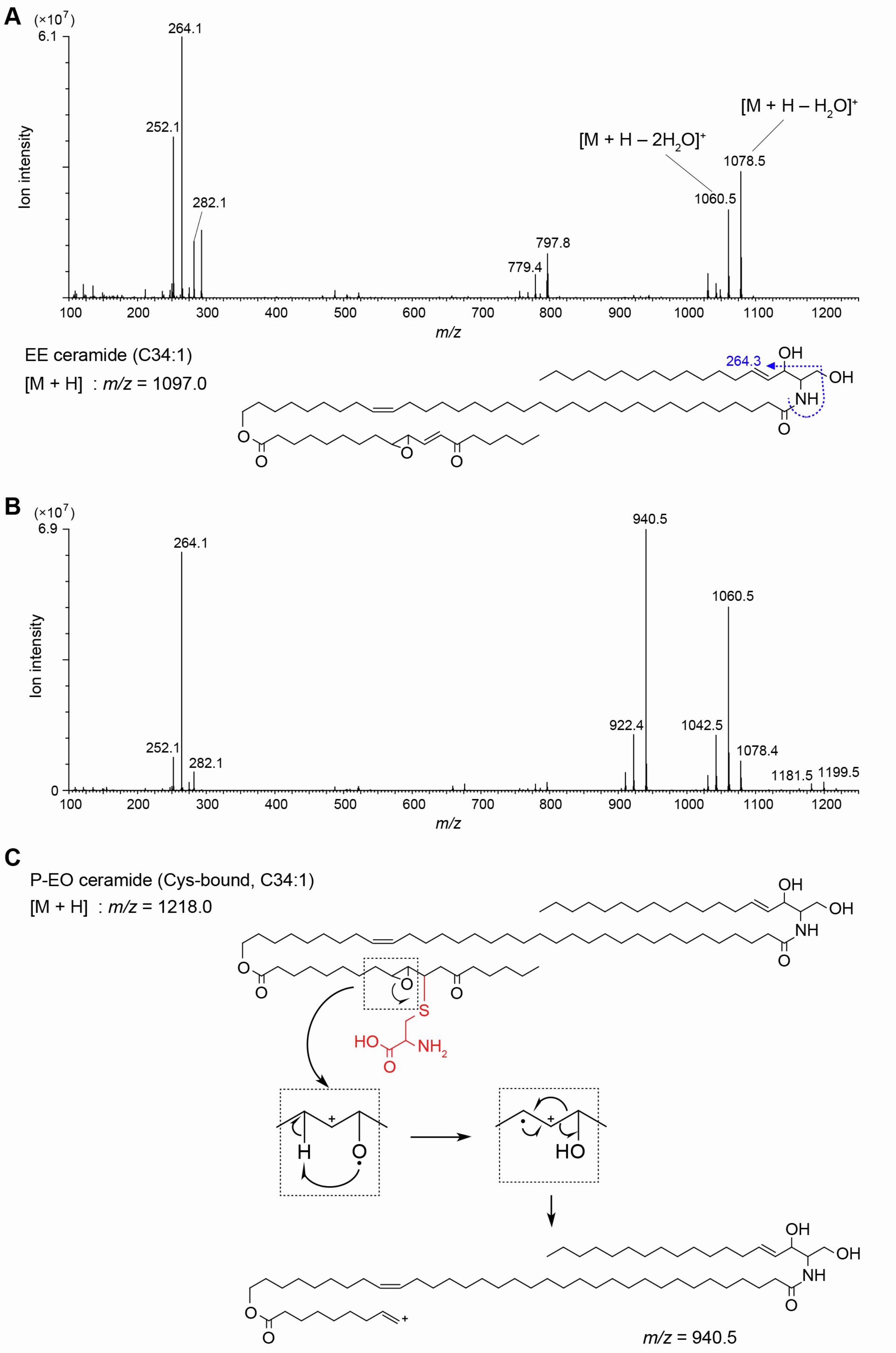

Figure S4. Product ion analysis of the EE ceramide–Cys conjugate, related to Figure 4.
(A–C) EE ceramides were incubated with 50 mM Tris-HCl (pH 7.4) (A) or Cys (B and C) at 37 °C for 1 h and subjected to LC-MS/MS analysis using product ion scanning mode. The proton adduct ion (*m/z* = 1,096.8) of EE ceramide containing C34:1 ω-OH FA (A) and the proton adduct ion (*m/z* = 1,218.0) of the corresponding EE ceramide–Cys conjugate (B) were selected as the precursor ions and the product ions were detected (scan range, *m/z* = 100–1,250). (C) The predicted fragmentation mechanism by which a product ion with *m/z* = 940.5 would be generated.

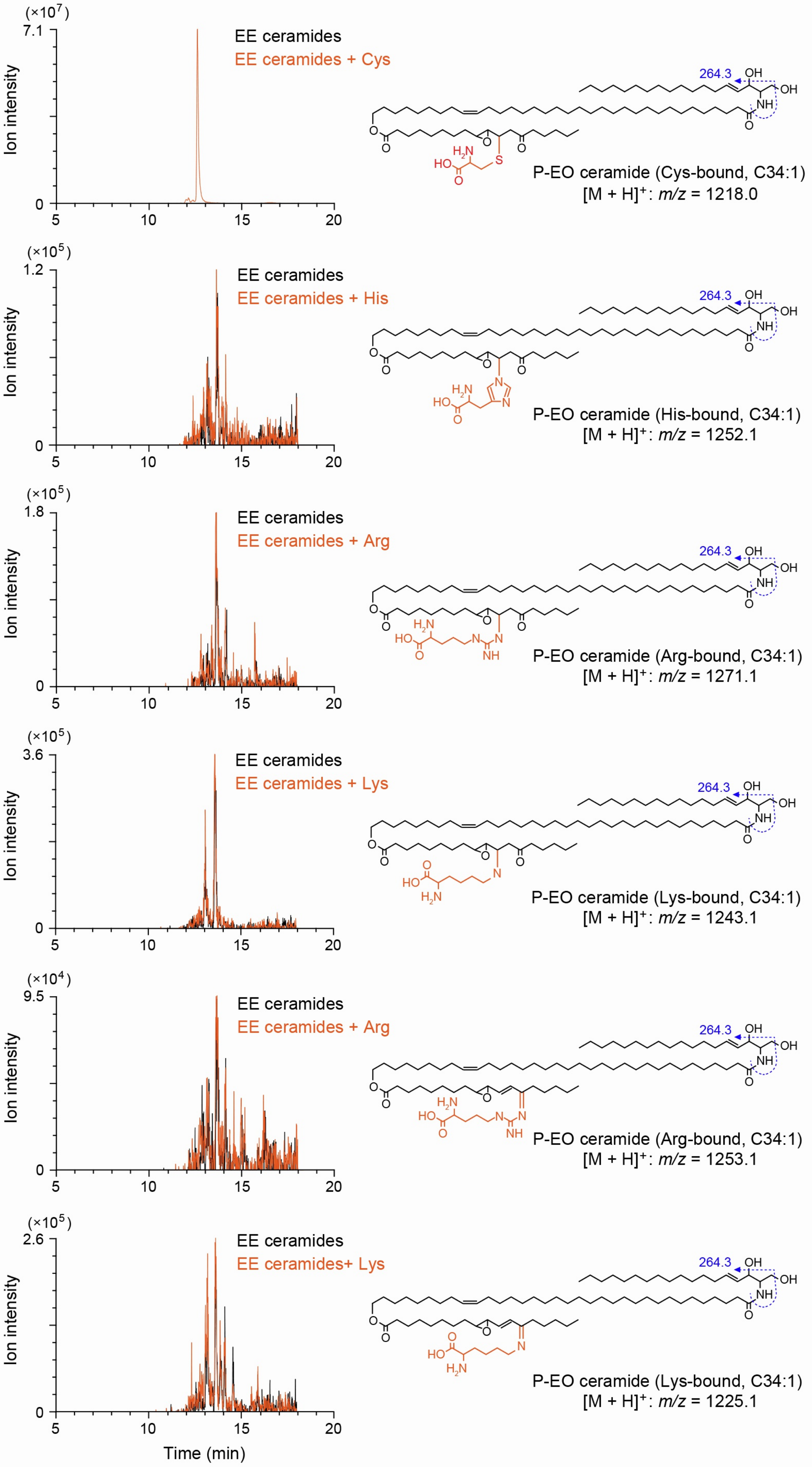

Figure S5. Detection of conjugates of EE ceramide and amino acid, related to Figure 4. EE ceramides were incubated with an amino acid (Cys, His, Arg, or Lys; 2 mM each) at 37 °C for 1 h and subjected to LC-MS/MS analysis using MRM mode. The proton adduct ions of each amino acid and EE ceramide containing C34:1 conjugates were selected as the precursor ions, and the product ion of *m/z* = 264 was selected.

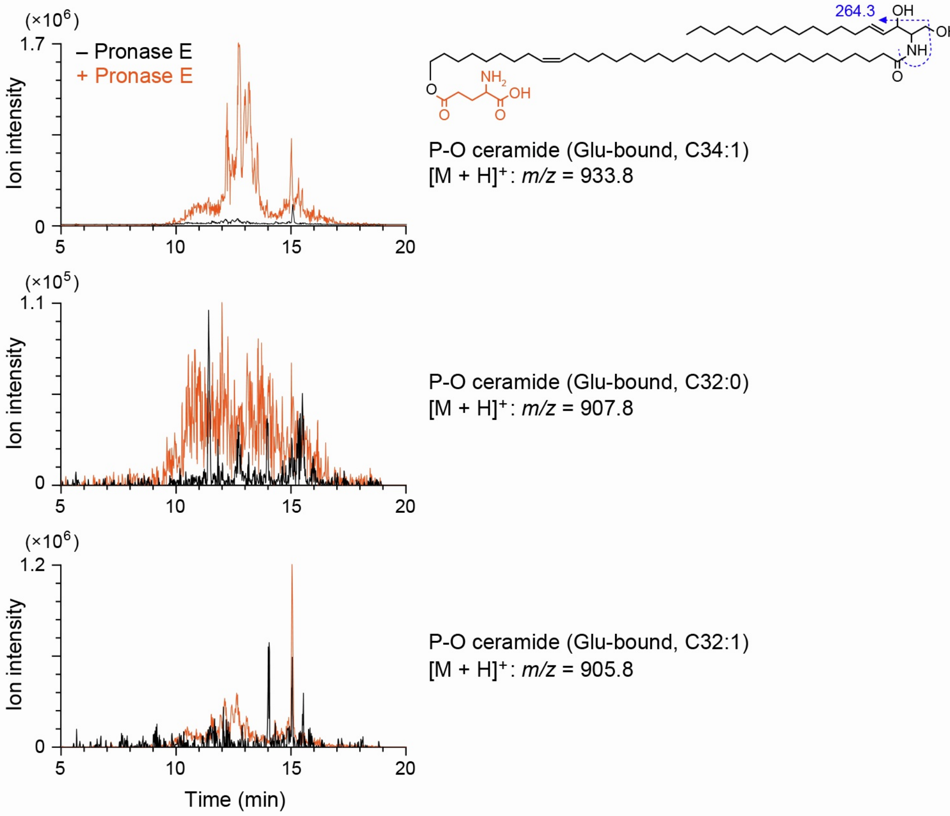

Figure S6. Absence of Glu-bound P-O ceramides in the epidermis, related to Figure 6. Protein-bound ceramide fractions prepared from WT and *Cyp4f39* KO mouse epidermis (10 mg) were digested with pronase E (1 mg/mL) at 37 °C for 2 h and subjected to LC-MS/MS analysis using MRM mode. The proton adduct ions of Glu-bound P-O ceramide containing C32:1, C32:0, or C34:1 *N*-acyl moiety were selected as the precursor ions, and the product ion of *m/z* = 264 was selected.

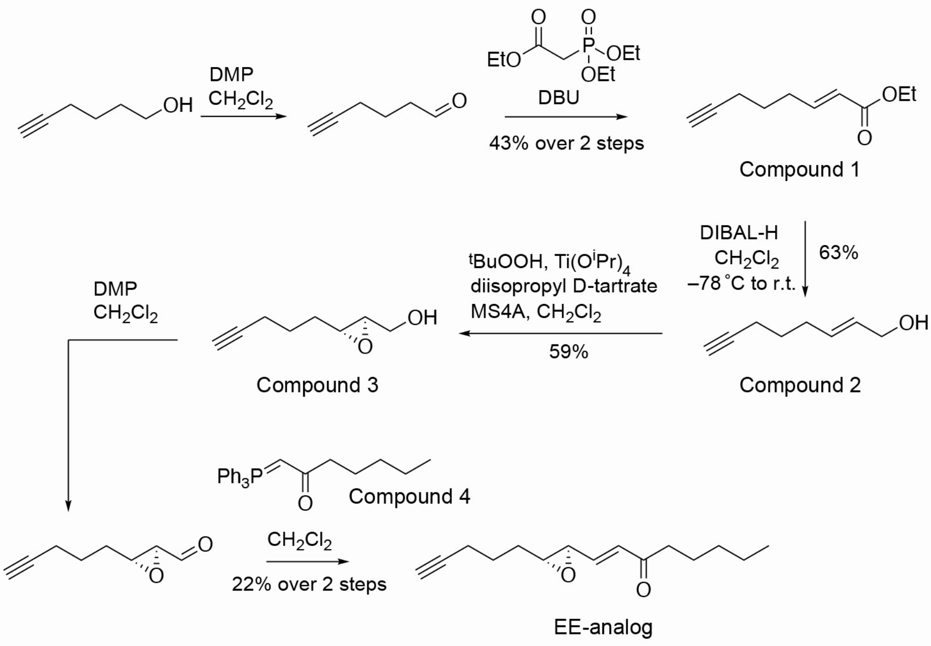

Figure S7. Scheme of EE-analog synthesis, related to Figure 2.
EE-analog was synthesized via the reaction scheme composed of six reaction steps as indicated. DMP, Dess-Martin periodinane; DBU, 1,8-diazabicyclo[5.4.0]undec-7-ene; DIBAL-H, diisobutylaluminium hydride; ^{t}BuOOH, *tert*-Butyl hydroperoxide; Ti(O^{i}Pr)$_4$, tetraisopropyl orthotitanate; and MS4A, molecular sieves 4A.

Table S1. MRM settings for detection of ceramide species in LC-MS/MS analyses.

Ceramides	*N*-acyl	Precursor ion (Q1)		Product ion (Q3)	Cone voltage (V)	Collision energy (eV)
		$[M + H – H_2O]^+$	$[M + H]^+$			
EE-ceramides	C30:0	1025.0	1043.0	264.3	30	35
	C30:1	1023.0	1041.0	264.3	30	35
	C32:0	1053.0	1071.0	264.3	30	40
	C32:1	1051.0	1069.0	264.3	30	40
	C34:0	1081.0	1099.0	264.3	30	40
	C34:1	1079.0	1097.0	264.3	30	40
	C36:0	1109.0	1127.0	264.3	30	45
	C36:1	1107.0	1125.0	264.3	30	45
EE-Cer–Cys conjugates	C30:0		1164.0	264.3	30	35
	C30:1		1162.0	264.3	30	35
	C32:0		1192.0	264.3	30	40
	C32:1		1190.0	264.3	30	40
	C34:0		1220.0	264.3	30	40
	C34:1		1218.0	264.3	30	40
	C36:0		1248.0	264.3	30	45
	C36:1		1246.0	264.3	30	45
ω-OH ceramides	C30:0	732.7	750.7	264.3	30	35
	C30:1	730.7	748.7	264.3	30	35
	C32:0	760.8	778.8	264.3	30	35
	C32:1	758.8	776.8	264.3	30	35
	C34:0	788.8	806.8	264.3	30	40
	C34:1	786.8	804.8	264.3	30	40
	C36:0	816.8	834.8	264.3	30	40
	C36:1	814.8	832.8	264.3	30	40
α-OH ceramides	d_9-C16:0	545.5	563.5	264.3	30	20
